# NLRP1- A CINDERELLA STORY: a perspective of recent advances in NLRP1 and the questions they raise

**DOI:** 10.1038/s42003-023-05684-3

**Published:** 2023-12-16

**Authors:** Kristian Barry, Christopher Murphy, Ashley Mansell

**Affiliations:** 1 Centre for Innate Immunity and Infectious Diseases, Hudson Institute of Medical Research, Clayton, VIC Australia; 2 Department of Molecular and Translational Sciences, Monash University, Clayton, VIC Australia; 3 7 Warren St., Upton, MA 01568 USA

**Keywords:** NOD-like receptors, Inflammasome

## Abstract

NLRP1, while the first inflammasome described, has only recently begun to gain significant attention in disease pathology, inflammation research, and potentially, as a therapeutic target. Recently identified human variants provide key insights into NLRP1 biology while its unique expression in barrier cells such as keratinocytes and airway epithelial cells has aligned with new, human specific agonists. This differentiates NLRP1 from other inflammasomes such as NLRP3 and identifies it as a key therapeutic target in inflammatory diseases. Indeed, recent discoveries highlight that NLRP1 may be the predominant inflammasome in human barrier cells, its primary role akin to NLRP3, to respond to cellular stress. This review focuses on recent studies identifying new human-specific NLRP1 mechanisms of activation of, gain-of-function human variants and disease, its role in responding to cellular stress, and discuss potential advances and the therapeutic potential for NLRP1.

## Abstract (web summary)

This mini review discusses recent discoveries in NLRP1 inflammasome biology, human specificity, its response to cellular stress and the implications for targeting inflammasome-mediated disease.

## Introduction

### NLRP1

NOD-like-receptor containing a Pyrin domain 1 (NLRP1) was the first described inflammasome-forming PRR to be discovered^1^. Despite this, our understanding and appreciation of the role and function of NLRP1 in immunity and disease have been restricted and underappreciated due to a lack of identified agonists, its limited expression and activation in myeloid cells and critically, its poor conservation between humans and rodents. Identification of Val-boroPro (Talabostat- a Dipeptidyl peptidase 8/9 inhibitor) as a dual mouse and human NLRP1 agonist^2,3^ provided the impetus and foundation for subsequent discoveries of human-specific NLRP1 ligands facilitating the expansion and understanding of the biochemistry and mechanisms of NLRP1 functionality (outlined below).

Like other NLRs, NLRP1 is structured with an N-terminal Pyrin domain, a NACHT and Leucine Rich Repeat (LRR), and a C-terminal CARD domains. NLRP1 also contains a function-to-find domain (FIIND) that is unique among NLRs, between its LRR and CARD domains. This allows the protein to undergo autoproteolysis, resulting in an N-terminal region and a C-terminal UPA-CARD domain that remain non-covalently associated in an inactive state^4^. Activation occurs when the N-terminal fragment is degraded by the proteasome and releases the UPA-CARD fragment which forms an active inflammasome complex^4–7^; an activation mechanism termed “functional degradation”. There is significant polymorphism between murine and human NLRP1, demonstrating a possible divergence in function during evolution. Indeed, while human NLRP1 is coded for by a single gene in humans, mice have three genes with up to six haplotypes (termed NLRP1a,b,c,d,e,f) with significant polymorphism within haplotypes depending on independent mouse strains^8,9^.

**Fig. 1:**
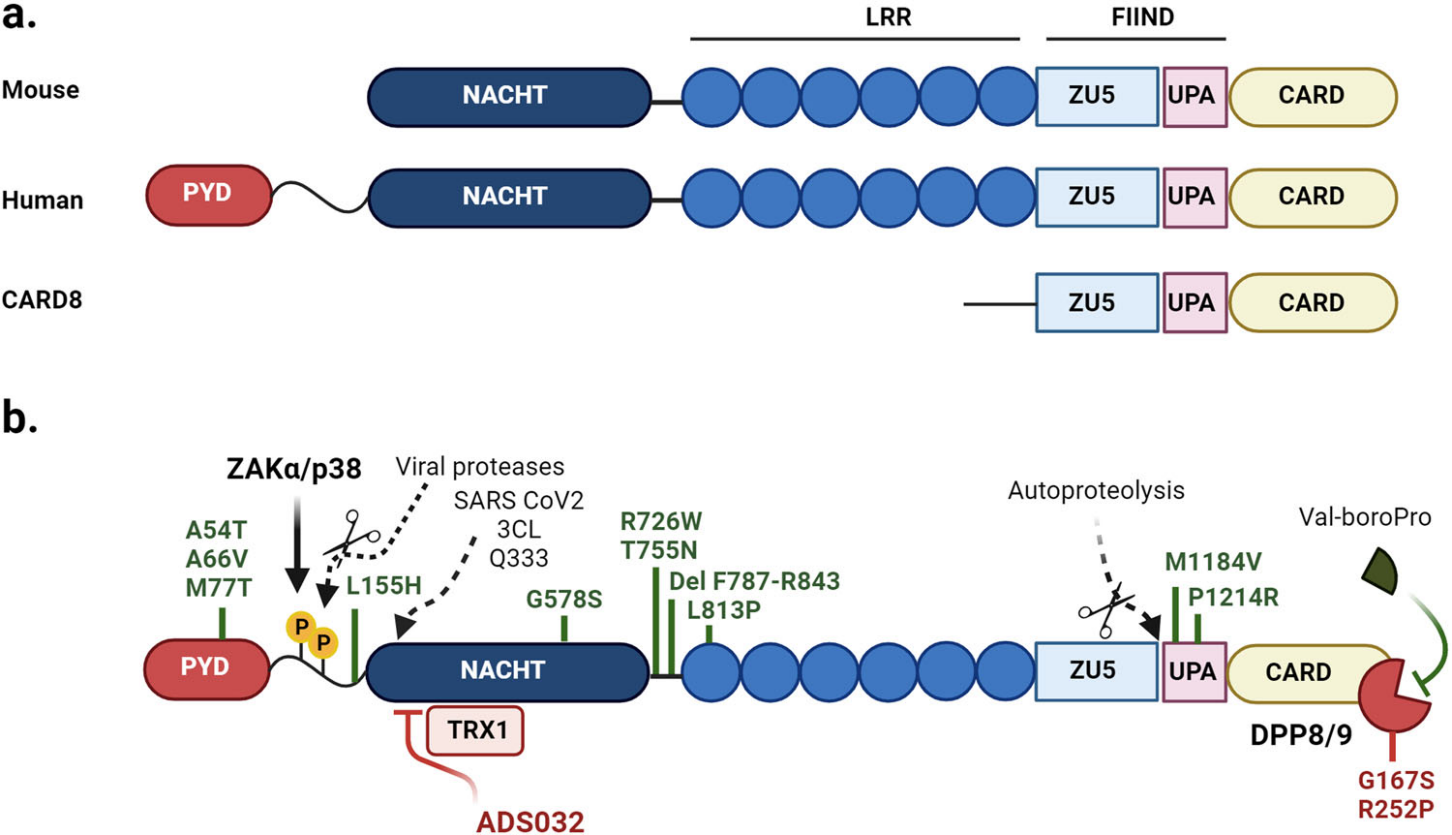
Schematic representation of NLRP1. **a** A comparison of architectural differences between mouse and human NLRP1. **b** Representation of the activating and restraining/inhibitory functions of NLRP1. Consistent between species, NLRP1 is constrained in an inactive form via DPP8/9 interaction with the FIIND domain, the inhibition of which by Val-boroPro (also called Talabostat) disengages DPP8/9 association inducing functional degradation and subsequent formation of an active inflammasome complex, while oxidized thioredoxin restrains NLRP1 activity via interaction with the NACHT-LRR domain. NLRP1 is also restrained by thioredoxin-1 (TRX1) which interacts with the NACHT/LRR domains while in response to ribotoxic stress, ZAKα and p38 MAP kinase induce phosphorylation of the disordered inter-region between the Pyrin and NACHT domains, mediating activation of NLRP1 and formation of a functional inflammasome complex. The small compound inhibitor ADS032 targets the Walker B motif within the NACHT domain to inhibit NLRP1 activation. The role of NLRP1 in disease is highlighted by gain of function mutations (marked in green: refer to Supplementary Table 1) in functional domains of NLRP1, while a loss of function variant in DPP8/9 (red) induces constitutive NLRP1 activity.

Thus, mouse and human NLRP1 share common architectural features such as the CARD, LRRs, FIIND domains as well as the central NACHT domain which contains Walker A and Walker B motifs responsible for ATP binding and hydrolysis, facilitating the structural transition of NLRs from an inactive to active moiety (Fig. 1). However, a critical variance is that human NLRP1 inflammasome oligomeric formation requires ASC recruitment to facilitate caspase-1 recruitment homologous to that observed with other NLRPs such as NLRP3^10,11^. Murine NLRP1 however, which lacks the PYD domain and directly recruits caspase-1 via its intrinsic CARD domain.

Therefore, while other inflammasomes such as NLRP3 display concordant ligands, mechanisms of activation (such as potassium efflux) and disease functionality across multiple species, the considerable deviation between mice and humans has limited the capacity to identify a role for NLRP1 in mouse models of disease that are conserved with human disease. As such, animal models of disease focussing upon, and identifying critical NLRP3 functionality may under-represent the role of NLRP1 in these diseases due to NLRP1 essentially being ‘inactive’ in the mouse due to structural and functional discrepancies. How this may impact upon the critical or central role of NLRP3 in many diseases in humans is yet to be understood.

### CARD8

While not the focus of this review, humans also express another closely related FIIND-containing PRR known as CARD8 that shares similar structure features to NLRP1 in that it can also undergo autoproteolysis and form an inflammasome, albeit without the requirement of ASC (Reviewed in ref. ^12^). While CARD8 is the only protein in humans that shares a FIIND-CARD domain with NLRP1, it lacks the LRR, NACHT, and PYD domains associated with NLRP1 (Fig. 1a). Furthermore, CARD8 is expressed in human but not murine systems, although unlike NLRP1, CARD8 is predominantly expressed in T cells^13,14^. CARD8 shares some similar activation mechanisms with NLRP1 in that it can be activated with Talabostat through DPP8/9 inhibition^15^, and has also been demonstrated to be activated by several viral proteases^16,17^. Indeed, the viral proteases from HIV-1 (dimerized gag-pol protease) and Coxsackie virus B3 (2A and 3C proteases) have both been identified to induce CARD8 activation and induce inflammasome formation in T-cells and endothelial cells respectively^16,17^. The activation of CARD8 through viral proteases draws similarities to the ‘tripwire immunity’ hypothesis regarding NLRP1 (detailed below), whereby CARD8 may act as a decoy protein “alarm” for viral proteases to elicit viral sensing.

### Expression of NLRP1 in humans

In humans, NLRP1 has been demonstrated to be found in the lining of the stomach, small intestine, colon, lung, endometrium and within keratinocytes of the skin^18–20^. The identification of gain-of-function mutations in human NLRP1 has provided key insights into the expression and pathology (see Supplementary Table 1), often leading to familial autoinflammatory disorders associated with skin and pulmonary inflammation. NLRP1 variants were first described in association with vitiligo alone or vitiligo-associated multiple autoimmune disease susceptibility^21^. Further studies by Zhong and Reversade identified that NLPR1 germline mutations caused skin inflammatory and cancer susceptibility spectrum diseases (multiple self-healing palmoplantar carcinoma and familial keratosis lichenoides chronica) highlighting that NLRP1 was not only highly expressed in keratinocytes but also the most prominent inflammasome in the skin^20^. A recent study identified for the first time two siblings sharing the same homozygous NLRP1 mutation but presenting with different clinical spectrum of severity^22^. While the less severe sibling presented with generalized inflammatory nodules with keratotic plugs resembling multiple keratocanthomas, the more severely affected sibling had manifestations of familial keratosis lichenoides chronica, psoriasis and atopic dermatosis, potentially identifying a role for NLRP1 in these diseases, supporting earlier NLRP1 genetic associations with psoriasis^23^.

A mutation in NLRP1 was also identified in systemic juvenile idiopathic arthritis patients resulting in a novel monogenic disorder autoinflammation with arthritis and dyskeratosis (AIADK)^24^, while a patient presenting with corneal intraepithelial dyskeratosis, mucosal inflammation, tooth abnormalities and eczema further expands the association of NLRP1 with autoimmune arthritis and eye disease^25^. Interestingly, Casanova et al.^26^ described an NLRP1 gain-of-function mutation in Juvenile-onset recurrent respiratory papillomatosis (JRRP) that leads to the growth of warts in the throat, respiratory distress and airway obstruction. JRRP is associated with infection with human papillomavirus of the α-genus and suggests and interplay between NLRP1, viral infection, and inflammation of pulmonary barrier cells. In addition, Masters and colleagues^27,28^ describe an NLRP1 variant (M1184V) within the FIIND domain that increases NLRP1 binding with DPP9 and is associated with asthma severity, further enhancing a role for NLRP1 in pulmonary inflammation. A study from the Peterlin group^29^ found a homozygous missense NLRP1 variant (G587S) in two siblings affected by concomitant multiple sclerosis and malignant melanoma, providing a potential genetic basis for association of NLRP1 not only with malignant skin cancer, but also neurodegenerative disease. box 1

Overall, these human genetic variants of NLRP1 highlight its role in a range of autoimmune and inflammatory disorders and identify NLRP1 as potentially the predominant inflammasome in barrier cells such as keratinocytes, corneal and bronchial epithelial cells. Therefore, in contrast to NLRP3 where genetic variants tend to manifest as systemic inflammatory syndromes, conventionally driven by myeloid cells, NLRP1-associated variant syndromes predominately manifest as inflammation of barrier cells and organelles. Further complicating translating murine mouse models into human disease, murine keratinocytes, unlike those of humans, lack or have very low NLRP1 inflammasome expression and are unable to respond to NLRP1 activators^19^. This lack of expression further complicates understanding the role of NLRP1 in murine disease models of inflammation.

Significantly, while NLRP1 is highly expressed in non-myeloid cells, we and others have found that pro-IL-1β and pro-IL-18 are constitutively expressed in barrier cells such as keratinocytes and bronchial epithelial cells^30–32^, while NLRP3 is absent. As such, these cells may not require priming to upregulated components of the inflammasome pathway to mediate inflammasome activation and maturation of IL-1β and IL-18 upon sensing stress or challenges potentially constituting a rapid response mechanism to external threats and disruptions to homeostasis as it is these barrier cells in the lung and skin that are the primary interactive cells extrinsic with exogenous or homeostatic challenges. Conversely, NLRP1 activation may provide the apex or priming ‘signal’ for myeloid cells and contribute to the inflammatory cascade in human disease.

Taken together, it could be hypothesized that based on genetic diseases and expression, while NLRP3 is the prevailing inflammasome in myeloid cells such as macrophages and dendritic cells, NLRP1 is the predominate inflammasome in barrier cells such as keratinocytes and epithelial cells. Whether NLRP1 and NLRP3 cooperate or coordinate these respective roles in maintaining homeostasis and immune response across tissue or specific cell types is yet to be determined.

Box 1 Facts and outstanding questions of NLRP1 in human biology **What we know**

- NLRP1 is a critical mediator of inflammation in barrier cells such as keratinocytes, bronchial epithelial and corneal cells.
- The identification of human NLRP1 and DPP9 variants identifies a direct role of NLRP1 in skin and pulmonary barrier cells autoimmune inflammatory diseases.
- NLRP1 demonstrates significant functional divergence between rodents and humans. Indeed, expression of the N-termini Pyrin domain required for inflammasome formation in humans is absent or non-homologous in many species.
- Species divergence, and lack of suitable animal models have significantly limited our understanding of the role of NLRP1 in disease.
- NLRP1 is activated by ZAKα, p38-induced phosphorylation in response to the ribotoxic stress response induced by a range of agonists.
- The recent discovery of multiple new NLRP1 human-specific agonists suggests NLRP1 may play a far more significant role in human disease than previously appreciated.
**What we need to know**
- While NLRP3 is the predominant inflammasome and driver of inflammation in myeloid cells, NLRP1 may be the principal inflammasome in non-myeloid cells, particularly where transcriptional regulation of inflammasome components such as pro-IL-1β, pro-IL-18 and NLRP3 are concerned.
- The identification of a suitable animal, or development of a “humanized” NLRP1 mouse, would allow the exploration of the role of NLRP1 in disease and the possible therapeutic targeting to address inflammatory diseases.
- The identification of an NLRP1 inhibitor would be a useful “tool” to further dissect the role of NLRP1 in human inflammatory models, analogous to MCC950 in exploring NLRP3 function.
- There may be redundancy amongst inflammasomes, with “cellular stress” recognized by both NLRP1 and NLRP3 inflammasomes, but reliant upon specific cellular expression.
- If there is redundancy, what are the implications for mono-inflammasome therapies currently under development to treat disease?

### DPP8/9 inhibition provides key mechanistic insights into NLRP1 function and activation

To date, the primary universal inducer of N-terminal degradation and subsequent inflammasome activation across both mice and humans are inhibitors of DPP8/9. Indeed, the disruption of DPP8/9 binding sites and catalytic activity by the chemotherapeutic drug Talabostat (also known as Val-boroPro) (reviewed in^12^), or by genetic knockout, induces NLRP1 inflammasome activation in both human and rodent cells^20,33^. DPP8/9 is an intracellular protease that cleaves after a proline residue at the penultimate P1 position via its dipeptidylaminopeptidase activity. While somewhat counterintuitive as to how targeting DPP8/9 aminopeptidase activity mediates both CARD8 and NLRP1 activation and inflammasome formation, subsequent studies identified that NLRP1 immunoprecipitated with DPP8/9^20^. Studies further identified that DPP9 interacts with the autoproteolytic FIIND domain of NLRP1, functioning to maintain NLRP1 in its inactive state. Displacement of DPP8/9 by Val-boroPro, releases NLRP1 and accelerates degradation of the N-terminal fragment, facilitating formation of an active NLRP1 inflammasome complex^33^.

Critically, human variant mutations in DPP9 (G167S) lead to NLRP1-dependent inflammasomopathies causing immune defects, recurrent fevers, bronchitis, susceptibility to Herpes infections, pancytopenia, anemia, and skin abnormalities^34^, consistent with NLRP1-mediated inflammation in barrier cells such as keratinocytes and bronchial epithelial cells. The recent identification of a patient displaying Hemophagocytic lymphohistiocytosis-like hyperinflammation presenting with skin manifestations, pancytopenia and increased susceptibility to infections was identified with a dominant-negative DPP9 mutation (R252P)^35^. This de novo mutation leads to destabilization of DPP9 and constitutive NLRP1 inflammasome activity, further supporting the concept that DPP9 ‘holds’ NLRP1 in an inactive state, lowering the threshold for NLRP1 activity.

While DPP8/9 inhibition ‘bridges the gap’ between human and mouse NLRP1, no physiologically occurring activator of NLRP1 in humans or mice has been shown to interact with DPP8/9. Therefore, it is likely that while humans and mice may share the same inhibitory complex, they are still activated by distinct and unique processes that target NLRP1 itself.

### NLRP1 and the sensing of stress

While research into NLRP1 was stymied for some time by the lack of bona fide specific activators, several recent studies have identified several human-specific activators of NLRP1 (see Supplementary Table 2).

#### Ribotoxic stress

While the trigger for mouse NLRP1 had been known for some time, one of the earliest described human activators of NLRP1 cells was UVB radiation, whereby irradiation of cells led to the induction of inflammasome activation and secretion of IL-1β, although the specific mechanism of activation was not yet described^36,37^. Subsequently in 2022, studies from the Zhong and Schmidt laboratories identified that UVB irradiation induced the ribosomal stress response in human keratinocytes and bronchial epithelial cells, leading to NLRP1 activation^32,38^. These studies demonstrated that UVB irradiation leads to RNA damage causing ribosomes to stall that activating the stress-activated protein kinases (SAP-kinases) p38, JNK and ZAKα^39,40^. ZAKα (and p38 to a reduced degree) then hyper-phosphorylates serine residues in the linker region of NLRP1 between the PYD and NACHT domains, leading to degradation of the N-terminal through a currently unknown pathway and allowing for the release of the UPA-CARD domain to form a functional inflammasome^32^. Furthermore, the same study identified the antibiotics Anisomycin, Hygromycin, and Doxyvinenol—known ribotoxic stress inducers^39,41^ as activators of ZAKα and subsequent NLRP1 agonists, further supporting the hypothesis that NLRP1 is a sensor for ribotoxic stress. Importantly, neither ribotoxic stress nor SAP-kinases were able to induce activation of murine NLRP1 as demonstrated via gene-supplementation studies, as murine NLRP1 lacks the linker domain found in human NLRP1 that undergoes serine hyper-phosphorylation, further highlighting the species specificity of NLRP1 activation.

Further leveraging the discovery that ribosomal stalling induced activation of NLRP1, two recent studies have proposed several new NLRP1 agonists based on their previously described capacity to induce ribotoxic stress. Robinson et al. identified that bacterial exotoxins including Diphtheria toxin from *Corynebactetrium diphtheriae*, Exotoxin A from *Pseudomonas aeruginosa*, and sidl from *Legionella pneumophila* that target the human ribosome elongation factors EEF1 and EEF2, inducing ZAKα, p38, and JNKs; triggering NLRP1-dependent pyroptosis in human keratinocytes^42^. Further complementing these studies, Pinilla et al. also describe ExoA, Diphtheria toxin, and Cholix toxin from *Vibrio cholerae* as targeting EFF2 to induce NLRP1 activity via ribotoxic stress and ZAKα/p38 in primary human corneal, nasal epithelial cells and the human alveolar epithelial cell line A549^43^, further developing the concept that NLRP1 is a critical sensor of cellular stress in non-myeloid cells such as keratinocytes, corneal and airway epithelial cells. Importantly in relation to disease pathogenesis, Pinilla and colleagues also described exacerbated p38 activity and IL-1β secretion and hypersensitivity to ExoA-induced ribotoxic stress-dependent NLRP1 inflammasome activation in nasal epithelial cells obtained from Cystic Fibrosis patients. These observations may, therefore emphasize the deleterious effects of *P. aeruginosa* infections of Cystic Fibrosis patients, which could also extend to Chronic Obstructive Pulmonary Disease patients who also suffer *P. aeruginosa* exacerbations and subsequent increased pulmonary inflammation and epithelial disruption.

#### Reductive and protein folding stress

Further enhancing the concept that NLRP1 is a stress sensor, recent studies from the Bachovchin group determined that protein folding stress and reductive stress both lead to NLRP1 activation, inflammation, and cell death^44–46^. Building on their earlier observation that oxidized thioredoxin-1 restrains NLRP1 activity^44^ in a manner somewhat reminiscent of DPP8/9 restraint upon NLRP1 activity, additional studies found that reductive stress abrogated this interaction and lead to inflammasome activation, accelerating proteasome-mediated degradation of the repressive N-terminal fragments of NLRP1, and releasing the inflammasome-forming C-terminal fragments from auto-inhibition^45,46^. Subsequent structural studies demonstrated direct interaction of thioredoxin with the nucleobinding domain of NLRP1 (see Fig. 1b), acting as a direct checkpoint on NLRP1 inflammasome activity^47^. Complementary studies, Bachovchin et al. found that small molecule inhibitors of metallo-aminopeptidases that target blockade of the N-end rule pathway, let to accumulation of proteosome-derived peptides and protein folding stress to accelerate N-terminal degradation NLRP1. However, this “trigger” alone was not sufficient to cause inflammasome activation as DPP8/9 effectively stabilized NLRP1 CT fragments, additionally requiring DPP8/9 binding ligands to overcome DPP8/9 checkpoint control of activation. Overall, it appears NLRP1 is maintained in a ‘ready’ state of activation, constrained like a primed spring by thioredoxin and DPP8/9 until released by these checkpoint inhibitors in response to stress.

### NLRP1 nuclei acid sensing

**Fig. 2:**
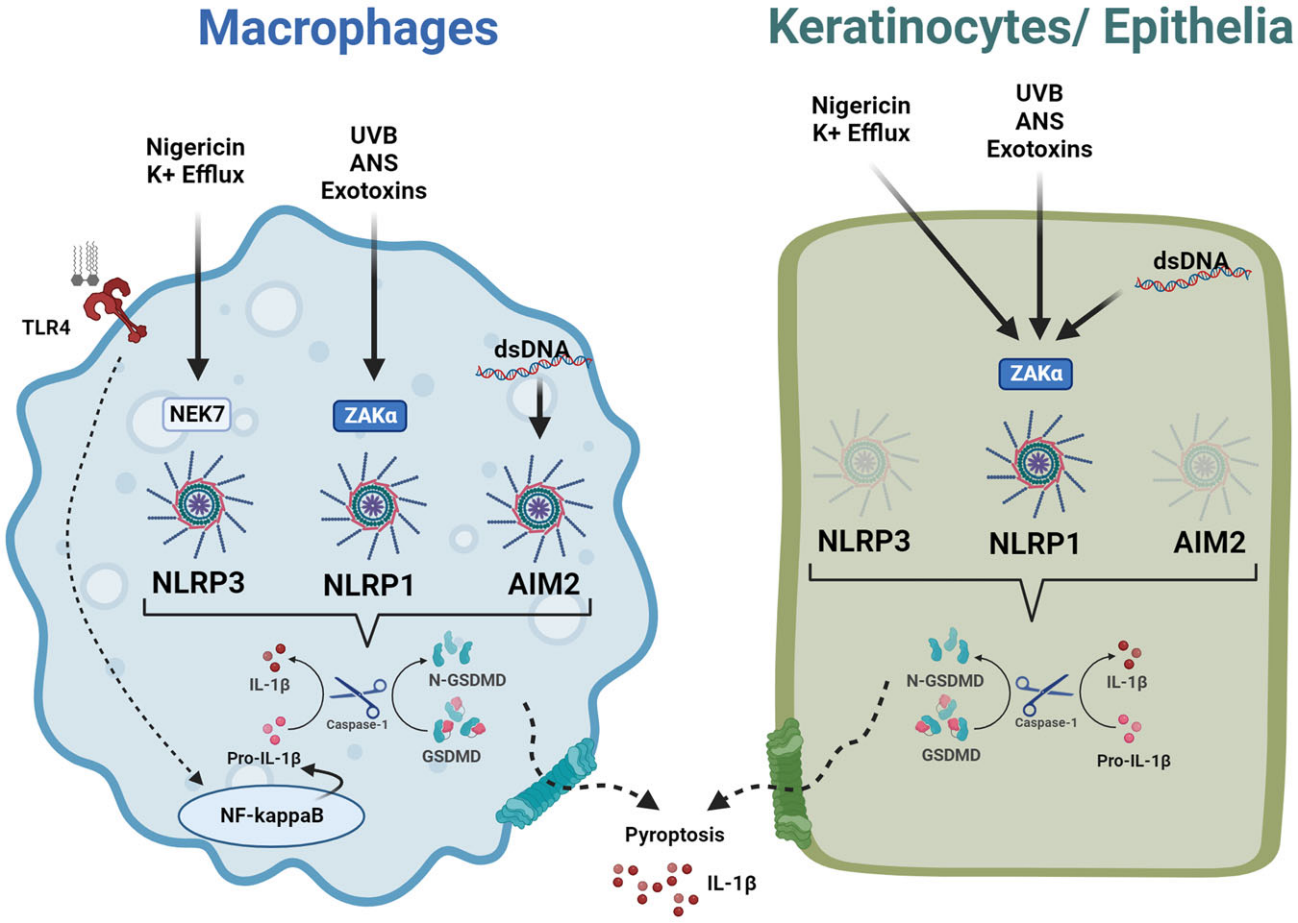
Cellular redundancy amongst inflammasomes converges upon NLRP1 in a cell-specific manner. While nigericin and dsDNA are considered prototypic NLRP3 and AIM2 in macrophages, in the absence of NLRP3 or AIM2, subsequent ribotoxic stress induces ZAKα-mediated activation of NLRP1. Created with BiorRender.com.

NLRP1 has also been shown to be activated by several viral products. Bauernfried et al. identified that Semliki Forest Virus activated NLRP1 in human keratinocytes, directly binding long stranded dsRNA poly(I:C)^30^. Subsequent NLRP1 activation was in part inhibited by the NLRP1 PYD domain which the authors hypothesized acts as a thresholder for dsRNA length. NLRP1 activity was recapitulated in primary human keratinocytes and bronchial epithelial cells, while reconstitution assays demonstrated that only human NLRP1, but not murine NLRP1b were responsive to dsRNA, identifying NLRP1 as a bona fide nucleic acid sensor. Further supporting the concept of NLRP1 as a nucleic acid sensor, a recent study from Fitzgerald and colleagues demonstrated dsDNA (poly dA:dT) -induced NLRP1 inflammasome activity in human keratinocytes^48^, in contrast to its well characterized binding and activation of the AIM2 inflammasome in myeloid cells. However, in contrast to dsDNA detection by cGAS-STING in human monocytes, human keratinocytes lack expression of AIM2, and dsDNA detection was independent of cGAS-STING. Studies identified that in human keratinocytes, poly (dA:dT) resulted in oxidative nucleic acid damage and cellular stress, activating ZAKα and subsequent NLRP1 activation via p38 hyperphosphorylation. Taken together, this non-canonical sensing would appear to be non-myeloid specific and in the absence of the canonical inflammasome sensor, in this instance AIM2, an alternative stress response converged on ZAKα-induced NLRP1 inflammasome activity (Fig. 2).

#### NLRP1 and viral “Trip-wire” immunity

Viral proteases are also able to cleave NLRP1 directly and lead to its functional degradation and subsequent activation, presumably circumventing the requirement for NLRP1 ligand binding or hyperphosphorylation^49^. A role for NLRP1 in sensing Human Rhinovirus, SARS-CoV2, Coxsackievirus, and a diverse range of Picornaviruses via viral proteolytic cleavage, and subsequent inflammasome formation has also been demonstrated. This function more closely resembles the function of murine NLRP1b, of which certain alleles can be cleaved by anthrax lethal toxin and cause inflammasome formation^8,50^. These findings suggest that NLRP1 viral sensing constitutes mammalian effector-triggered immunity (ETI) or “trip-wire” immunity, which detects the activity of pathogen-encoded effectors, such as toxins and proteases (reviewed in ref. ^51^) and initiates “functional degradation” and subsequent inflammasome activation. While not constituting traditional pattern-associated molecular patterns (e.g., all RNA viruses generate dsRNA), pathogen-encoded activities sensed by ETI sense factors important for pathogen fitness and are therefore evolutionarily constrained allowing ETI sensors to detect viable or replicating pathogens without the need of conservation. Curiously, Daughtery et al. identify that the picornaviridae protease responsible for cleaving NLRP1 within the rapidly evolving linker region between the PYD and NACHT domain, coincidently the same region that undergoes ZAKα/p38-induced hyperphosphorylation^49^. Whether these two events are related, due to evolutionary pressure or coincidence, is yet to be determined.

A recent study by Jenster et al. has further identified alphavirus and anisomycin induction of NLRP1 activation through p38, which directly phosphorylates NLRP1 in order for it to activate^38^. Interestingly, this study found that ZAKα accounted for a significant, but not all, activation of NLRP1 through knockout experiments in keratinocytes, and the authors suggest that their model of viral induction of NLRP1 is independent of ribosomal stress. However, the involvement of SAP-Kinases in both ribotoxic and viral settings may suggest pathway convergence between agonists.

Taken together, therefore, it may be proposed that akin to potassium efflux activation of NLRP3 as a common response mechanism to stress in myeloid cells, ribotoxic stress and the subsequent ZAKα-mediated NLRP1 activation is a common response to extrinsic stress inducers in non-myeloid cells. Intriguingly, a recent study from Zhong and colleagues report that the prototypic NLRP3 agonist nigericin activates NLRP1 in primary human skin, nasal and corneal epithelial cells^52^. NLRP1 activation by nigericin requires K^+^ efflux-driven ribosome stalling and the ribotoxic stress response sensor, ZAKα. Alternate NLRP1 sensing of K^+^ efflux occurs due to the absence of NLRP3 expression in keratinocytes and epithelial cells (which constitutively express pro-IL-1β and IL-18, thus not requiring priming) (Fig. 2), suggesting that cellular stress is a common activator of inflammasomes, the specificity of which is dependent on cell type and sensor expression.

Ribotoxic stress response is activated by a sub-set of oft used anticancer drugs such as Doxorubicin and Daunorubicin that are associated with inducing inflammation (reviewed in ref. ^41^). In addition, Ricin from *Riciunus communis* and Shiga toxin from *Shigella are also associated with inducing the ribotoxic stress response and ZAKα activation*, while ribotoxic stress is also implicated in the pathology of Huntingtin’s Disease^53,54^. It will be interesting to see if future studies identify a role for NLRP1 in the pathologies associated with these treatments and diseases. The disordered region between the PYD and NACHT domain of NLRP1 is the target of ZAKα-mediated hyperphosphorylation, and appears under strong positive selection as described above. NLRP1 may, therefore be at the forefront of the evolutionary arms race between pathogens and humans, potentially emphasizing the critical role NLRP1 in maintaining health and homeostasis.

### Outlook and perspective

While NLRP1 was the first described inflammasome, it has remained somewhat of an enigma in the inflammasome field, relegated to the periphery of inflammasome biology. Despite this however, NLRP1 has been implicated in a wide range of diseases such as cardiovascular, neurodegenerative, intestinal, ocular and pulmonary diseases, similar to that described and characterized for NLRP3. Two major factors have impacted our understanding and development of NLRP1 biology; (1) divergence between mice and humans significantly restricting investigating NLRP1 in disease models, and (2) the lack of bona fida NLRP1 agonists and antagonists to dissect NLRP1 function.

Recent breakthrough studies, however, have realigned our understanding of NLRP1 function. The discovery and characterization of human variants with inflammasomopathies have provided insights into the biological outcomes, and potential disease phenotype, of dysregulated NLRP1-induced inflammation. Notably, the identification of stress responses such as ribotoxic stress converging on ZAKα-mediated signaling, protein folding and reductive stress inducing NLRP1 activity allow speculation that parallel to that observed for NLRP3, NLRP1 response to stress is an analogous immune reaction, as sensors of disruptions to homeostasis. The divergence between NLRP1 and NLRP3 may lie however, in the cell types in which each inflammasome is active; be it myeloid for NLRP3 and non-myeloid for NLRP1, the difference aligning with tissue specific expression of each inflammasome and the contribution of each, either in combination or individually in a diseased organ. Moreover, it could be hypothesized that there may be redundancy amongst inflammasomes, consistent with that observed amongst other pattern recognition receptors such as Toll-like receptors (TLRs)^55,56^. NLRP1 is activated by ZAKα in response to prototypic NLRP3 (nigericin) and AIM2 (poly (dA:dT)) agonists in cells that lack NLRP3 expression. NLRP1 may, by default, become the surrogate sensor of cellular stress, albeit with delayed kinetics. Conversely, this may raise the possibility therefore, that targeting of NLRP3 with highly efficacious inhibitors could inadvertently induce proxy activation of NLRP1 and maintain an inflammatory environment.

Consequently, could the supposition of the prominent role of NLRP3 in so many diseases, and the therapeutic strategies aligned with this, have overlooked a potential role for NLRP1 due to species discrepancies and the heavy reliance, and concordant NLRP3 biology, of disease models in mice? To address these questions, the development of a ‘humanized’ NLRP1 mouse, or alternative model systems may prove transformative in our understanding of the role of NLRP1 and inflammasomes, in disease phenotypes. The discovery of the specific NLRP3 small molecule inhibitor MCC950 significantly enhanced investigating NLRP3 function, pathophysiology, and therapeutic potential^26,57^. The recent description of a novel, dual NLRP1 and NLRP3 inhibitor called ADS032^31^, may provide a useful tool to investigate NLRP1 inflammasome biology and explore the therapeutic potential of targeting NLRP1. Additionally, future studies in human organotypic 3D models of disease or clinical isolates may provide key insights into the role of NLRP1 in diseases.

In conclusion, while NLRP1 has for so long been the wallflower at the inflammasome dance, it is apparent that considerable research is still to be conducted and much to learn in this ever-evolving field, such that one day, NLRP1 may well become the Belle of the Ball.

### Supplementary information

Supplementary Tables
