# Supplementary material for: NLRP1- A CINDERELLA STORY: a perspective of recent advances in NLRP1 and the questions they raise
Source: Commun Biol. 2023 Dec 16;6:1274. doi: 10.1038/s42003-023-05684-3 (PMC10725483; doi:10.1038/s42003-023-05684-3)
Supplement: Supplementary file 1 — Supplementary Tables [file 42003_2023_5684_MOESM1_ESM.pdf]

Supplementary Tables

NLRP1- A CINDERELLA STORY: a perspective of recent advances in NLRP1 and the questions they raise.

Kristian Barry [1,2], Christopher Murphy [3], and Ashley Mansell [1,2]

[1]Centre for Innate Immunity and Infectious Diseases, Hudson Institute of Medical Research, Clayton, VIC, Australia
[2]Department of Molecular and Translational Sciences, Monash University, Clayton, VIC, Australia
[3]7 Warren St., Upton, MA 01568, USA

Corresponding Author:

Ashley Mansell: ashley.mansell@hudson.org.au

Mutation	Region	GoF/LoF	Disease	Phenotype	Ref.
**A54T**	PYD	GoF	Multiple self-healing palmoplantar carcinoma (MSPC), familial keratosis lichenoides chronica (FKLC).		20
**A66V**	PYD	GoF	MSPC, FKLC.		20
**M77T**	PYD	GoF	MSPC, FKLC, Corneal intraepithelial dyskeratosis.	Corneal lesions and thickening	20, 58
**L155H**	Linker (PYD-NACHT)	GoF	Vitiligo, autoinflammatory disease, COVID-19 severity*	Increased IL-1β processing.	21, 59, 60
**G578S**	NACHT	GoF	Multiple sclerosis and malignant Melanoma.	Vertigo, weakness of limbs, sight issues, melanoma. Increase gene expression in IL-1β, NFKB1, JUN and P38 pathways.	29
**p.R726W**	Linker (NACHT-LRR)	GoF	NAIAD – NLRP1-associated autoinflammation with arthritis and dyskeratosis.	Recurrent fever, dyskeratosis, increased Neutrophils. IL-18 and cleaved caspase-1	24
**T755N**	Linker (NACHT-LRR)	GoF	Juvenile onset recurrent respiratory papillomatosis.	Laryngeal lesions, papilloma growth, warts, mild keratosis pilaris.	61
**L813P**	LRR	GoF	Differed between siblings, one with Multiple keratoacanthomas, one with familial keratosis lichenoides chronica.	Erythema, anaemia, blistering, eye inflammation, hyperkeratotic plugs, papillomatosis, failure to thrive.	22
**p.F787_R843del**	LRR	GoF	MSPC, FKLC.		20
**M1184V**	FIIND	GoF	Asthma*, Vitiligo.	Increased IL-1β processing.	27, 60
**P1214R**	FIIND	GoF	NAIAD – NLRP1-associated autoinflammation with arthritis and dyskeratosis.	Recurrent fever, dyskeratosis, increased Neutrophils. IL-18 and cleaved caspase-1	24
**G167S**	DPP9	LoF (in DPP9)	Hatipoğlu syndrome.	Recurrent HSV infection, airway inflammation, skin pigmentation anomalies.	34
**R252P**	DPP9	LoF (in DPP9)	Hemophagocytic lymphohistiocytosis–like hyperinflammation	Pancytopenia, skin manifestations, increased susceptibility to infections	35
**R147C, Asn864ThrfsTer4 (frameshift), T246S, T878M, T995I, M1119V, V1241L.**		N/A	Multiple Sclerosis*		29
**rs12150220**	N/A	N/A	Type 1 diabetes*		62
**rs8079034C and rs878329C**	N/A	N/A	Psoriasis*		23
**rs2137722/G-rs12150220/T-rs2670660/G**	N/A	N/A	Leprosy*	Genetic association with SNPs, if missing rs2137722 then only associated with one form of leprosy.	63
**rs2670660, rs12150220-rs2670660**	N/A	N/A	Systemic Lupus*	Increased rash, nephritis and arthritis	64
* Genetic association disease studies					

Supplementary Table 1. Summary of NLRP1 gain of function variants and their disease associations.

Activator	Human / Mouse	Mechanism	Ref.
**Val-BoroPro / Talabostat**	Mouse and Human	Inhibits DPP9 inhibitory complex	[3, 33, 65]
**Anthrax Lethal Factor**	Mouse	Functional degradation	[7, 8]
**Toxoplasma Gondii infection**	Mouse	N/A	[66, 67]
**UVB Radiation**	Human	Ribotoxic Stress	[32, 36, 37]
**Anisomycin**	Human	Ribotoxic Stress	[32]
**Hygromycin**	Human	Ribotoxic Stress	[32]
**Doxyvinenol**	Human	Ribotoxic Stress	[32]
**Diptheria toxin**	Human	Ribotoxic Stress	[42, 43]
**Exotoxin A (pseudomonas)**	Human	Ribotoxic Stress	[42, 43]
**Sidl (Legionella)**	Human	Ribotoxic Stress	[42]
**Cholix toxin**	Human	Ribotoxic Stress	[43]
**Nigericin**	Human	Ribotoxic Stress	[52]
**dsRNA (Poly(I:C))**	Human	Direct Sensing	[30]
**dsDNA (Poly(dA:dT))**	Human	Direct Sensing	[48]
**Viral 3C Proteases**	Human	Protease activity - Tripwire	[49]
**Alphavirus**	Human	Ribotoxic Stress	[38]

Supplementary Table 2. Summary of NLRP1 agonists and species specificity
